# Preventive measures for the progression of dysphagia in patients with cancer of head and neck subjected to radiotherapy: a systematic review with meta-analysis

**DOI:** 10.1590/2317-1782/20232021246en

**Published:** 2023-05-01

**Authors:** Amanda Guterres Beuren, Émille Dalbem Paim, Nathália da Silva Flores, Vera Beatris Martins, Fabricio Edler Macagnan

**Affiliations:** 1 Universidade Federal de Ciências da Saúde de Porto Alegre - UFCSPA - Porto Alegre (RS), Brasil.; 2 Irmandade Santa Casa de Misericórdia de Porto Alegre - ISCMPA - Porto Alegre (RS), Brasil.; 3 Departamento de Fisioterapia, Universidade Federal de Ciências da Saúde de Porto Alegre - UFCSPA - Porto Alegre (RS), Brasil.

**Keywords:** Prophylaxis, Preventive Measures, Dysphagia, Head and Neck Neoplasms, Radiotherapy

## Abstract

**Purpose:**

To identify the effects of prophylactic, non-pharmacological measures on the progression of dysphagia in patients with head and neck cancer undergoing radiotherapy.

**Research strategies:**

The search was performed in Medline (via PubMed), Scopus, and Embase databases, as well as in the gray literature.

**Selection criteria:**

Randomized clinical trials were included, with adult patients (≥ 18 years old) and diagnosed with head and neck cancer, treated with radiotherapy (with or without surgery and chemotherapy), and submitted to non-pharmacological protocols for the prevention of dysphagia.

**Data analysis:**

The risk of bias was assessed using the PEDRO scale and the overall quality of evidence was assessed using the GRADE instrument.

**Results:**

Four studies were considered eligible, and of these, two were included in the meta-analysis. The result favored the intervention group, with a mean difference of 1.27 [95% CI: 0.74 to 1.80]. There was low heterogeneity and the mean score for risk of bias was 7.5 out of 11 points. The lack of detail in the care with selection, performance, detection, attrition, and reporting biases contributed to the judgment of the quality of the evidence, considered low.

**Conclusion:**

Prophylactic measures to contain dysphagia can promote important benefits on the oral intake of patients with head and neck cancer when compared to those who did not undergo such a therapeutic measure during radiotherapy.

## INTRODUCTION

Dysphagia is a common alteration in patients with head and neck cancer (HNC)^(1)^. This disorder causes oral nutrition limitation and damage to the nutritional state, increasing the risk of recurrent aspiration pneumonia^(2)^. Due to the role of oral nutrition in the functionality and quality of life, it is fundamental to detect early alterations indicating the onset of dysphagia to soften the impact of the disorder on the cancer treatment course^(3)^.

It is known that patients with HNC who are subjected to radiotherapy (RT) may have their swallowing function more damaged than those who are only subjected to surgical intervention. The adverse effects caused by radiation, like mucositis, xerostomia, pain, skin reactions, and swelling, combined with tissue fibrosis, contribute to swallowing deterioration^(4)^.

In general, the whole skeletal muscle is affected by the poor nutritional state caused by the reduction in swallowing capacity. In such a process, the muscle involved in the swallowing process loses performance and the clinical condition worsens^(5,6)^. In these cases, it is common to resort to alternative nutrition routes during the treatment; however, it is known that despite being necessary, an artificial diet can be insufficient to maintain the nutritional state and may also act negatively on the evolution of dysphagia^(7-9)^.

Due to the adverse effects that appear throughout the treatment, the individuals may have their oral food intake either limited or interrupted, and such disuse of the muscle involved in swallowing can stimulate the remodeling of the muscles and possibly potentialize fibrosis and radio-induced swelling^(5,6)^. The skeletal muscles start to show evidence of atrophy by disuse only a few hours after immobilization^(5,10)^.

Dysphagia recovery associated with HNC involves multiple approaches, especially in patients treated with radiotherapy due to the long-term effects induced by ionizing radiation (tissue fibrosis). Over the past few years, the effect of different prophylaxis models on dysphagia has been investigated in the context of antineoplastic treatment to identify the ideal moment to start an intervention^(9,11)^.

These are some important measures since RT, despite its desired antitumor effects, promotes cumulative effects in molecular routes of the skeletal muscle, which implies changes in the muscle configuration with mutation of both the type and size of the muscle fibers, an increase of local fatty tissue, and redistribution of fibers in the muscle^(5,6)^.

Even though these preventive measures have been investigated and sometimes applied, a consensus is yet to be reached regarding the effect of prophylactic measures on the degree of dysphagia. Therefore, this systematic review study aimed to assess the effect of prophylactic interventions on the progression of dysphagia associated with HNC in patients subjected to radiotherapy to guide and favor the decision-making process in the early clinical management^(12)^.

## PURPOSE

This study aimed to identify the effect of non-pharmacological prophylactic measures on the progression of dysphagia in patients with head and neck cancer who are subjected to radiotherapy.

## RESEARCH STRATEGIES

This study is based on the recommendations of the Cochrane Handbook^(13)^. The review is described according to the Preferred Reporting Items for Systematic Reviews and Meta-Analyses (PRISMA Statement) according to the checklist in the Supplementary Material^(14)^. This research was also registered in the International Prospective Register of Systematic Reviews (PROSPERO) identified as CRD42021226726, using the PICO (Population, Intervention, Comparison/Control, Outcome) strategy^(15,16)^.

## SELECTION CRITERIA

We included only randomized clinical tests whose at least one arm analyzed the prophylactic effect of non-pharmacological interventions on dysphagia by comparing the results with a control group. The sample includes adult patients with a diagnosis of HNC and indication of radiotherapy, associated or not with chemotherapy and surgery, with or without dysphagia at the beginning of the study.

We considered the following primary outcome: progression of dysphagia degree assessed by the difference between the initial and final assessments. There were no restrictions regarding the dysphagia measurement method or instrument since the results were normalized through the difference between the initial and final degrees of the dysphagia state. The secondary outcome considered the analysis of the nutritional profile and the presence of alternative nutrition routes.

As for the intervention of interest, there was no restriction or referring to either one or another prophylactic measure of prevention or progression (worsening) of dysphagia. The techniques of specific prophylaxis were not compared, only their effects were measured regarding the usual care (usually based on guidelines) or regarding a placebo or sham treatments.

We excluded studies without any non-pharmacological intervention for the prevention of dysphagia and/or speech therapy assessment.

Our search was performed on the following main databases: Medline (via PubMed), Scopus, and Embase, in addition to the gray literature on the Clinical Trial, WHO International Clinical Trials Registry Platform, REBEC, OpenGrey, as well as abstracts of potentially relevant congresses over the past five years. A manual search for papers was also conducted by screening the references of the papers included in this systematic review. We started the selection process of the studies right after the last search (December 2020).

There was no restriction of languages or publication dates for the studies on the databases and in the gray literature. We used several morphological variations and synonym terms related to the following words: “Head and neck neoplasms”, “Deglutition disorders”, “Dysphagia”, “Swallowing disorders”, “Prevention”, “Prophylactic”, “Randomized controlled trial”, “randomized”, “controlled”, and “trial”. The full search strategy for each bibliographic base is described in the Supplementary Material.

After excluding the repeated papers, two reviewers (AGB and NSF) assessed the titles and abstracts independently. The papers were selected based on the eligibility criteria using the software of bibliographical management (Mendeley). At this step, any disagreements were analyzed by a third reviewer (FEM).

After the exclusion based on the titles and abstracts, the full texts were read by the two reviewers for the final decision of either including or excluding the paper. Any disagreements were resolved by a third reviewer (FEM).

Data extraction was performed by two authors (AGB and NSF) using a standard form for the following information: study design, first author, year of publication, location, sampling size, clinical characteristics of the volunteers, detailed description of the interventions implemented, control groups, and pre-and post-treatment values for the results generated from the different dysphagia assessment scales, both for the control group and the intervention group.

All information was organized and stored in a file on the Excel software, and the disagreements between the authors were resolved by consensus with the third reviewer, who performed the data checking.

## DATA ANALYSIS

Two independent reviewers (EDP and VBM) assessed the risk of bias (PEDRO scale)^(17)^, and the final evaluations were discussed and defined combined with all authors. To score the criteria of the scale, the information must be clear and objective, otherwise, the score is considered null. Chart 1 shows the respective results.

**Chart 1:** Assessment of the methodological quality of the papers according to the Pedro scale^17^

PEDRO CLASSIFICATION / PAPERS		Carnaby-Mann et al.^(11)^	Messing et al.^(18)^	Mortensen et al.^(19)^	Kotz et al.^(20)^
External Validity	1 Inclusion criteria	Y	Y	Y	Y
(Max = 1)					
Internal Validity	2 Random allocation	Y	Y	Y	Y
	3 Secret allocation	N	Y	N	N
	4 Similar group at the start of the study	Y	Y	Y	Y
	5 Blinding of the participants	N	N	N	N
	6 Blinding of the therapists	N	N	N	N
(Max=8)					
	7 Blinding of the assessment	N	Y	N	N
	8 Analysis of 86% of the sample	Y	Y	Y	Y
	9 Analysis of the treatment goal	Y	Y	Y	Y
Interpretation of the outcomes	10 Comparison between groups	Y	Y	Y	Y
	11 Measures of central and dispersion trend	Y	Y	Y	Y
(Max=2)					
Total of scores	-	7	9	7	7
(Max = 11)					

Caption: Y = yes; N = no; Max = maximum

The global quality of evidence was assessed based on the GRADE approach^(21,22)^. For each outcome, the quality of evidence is initially considered ‘high’ and subsequently can be lower graded to the levels of ‘moderate’, ‘low’, or ‘very low’ quality, depending on the assessment of the following five criteria: risk of bias in the individual studies, indirect evidence, heterogeneity, imprecision, and risk of bias in the publication. The quality of evidence was individually evaluated in two ways: a) for the body of evidence composed only of studies included in the meta-analysis and b) for the body of evidence included in this systematic review, composed of the entirety of the narratively synthesized individual studies^(22)^.

The bias of publication was assessed through linear regression of the estimates of the intervention effect by its reverse variance using the Egger test and a Funnel Plot chart.

**Figure 1:**
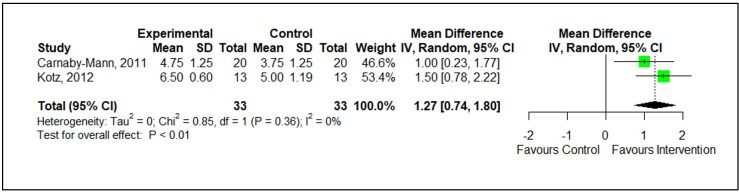
Meta-analysis of the prophylactic effect on the progression of dysphagia in patients with cancer of the head and neck subjected to antineoplastic treatment. The degree of dysphagia was assessed through the FOIS scale in both studies with a similar period of comparison - 6 weeks (10) and 3 months (20) - to reduce the gap between the studies

According to Figure 1, only the results from two studies^(11,20)^ were statistically collected from a meta-analysis. The analysis followed the reverse method of variances and estimator of Der Simonian and Laird for τ^2^ in a model of random effects, which allows for statistically incorporating the variability between studies into the estimate of the final effect. For continuous outcomes, we used the data of the post-treatment means of each group to calculate the effect size (Cohen D) from a mean weighted difference (MWD).

The results of studies that did not report the data as mean and standard deviation (SD) in the metanalyses were included by converting the data from median to mean according to the Hozo method^(15)^. All analyses were performed on the RStudio software (version 1.3.1093) using the ‘meta’ package in the R language (version 4.0.3).

The statistical heterogeneity was quantitatively assessed using the *I^2^* statistical and the *χ^2^* test. The statistical heterogeneity was interpreted according to the most recent guidelines (Cochrane Handbook, version 6.0)^(13)^. Heterogeneity is classified based on the *I^2^* values as follows: up to 40% is a trivial effect, from 30 to 60% is moderate, from 50 to 90% is substantial, and from 75% to 100% is considerable heterogeneity.

## RESULTS

**Figure 2:**
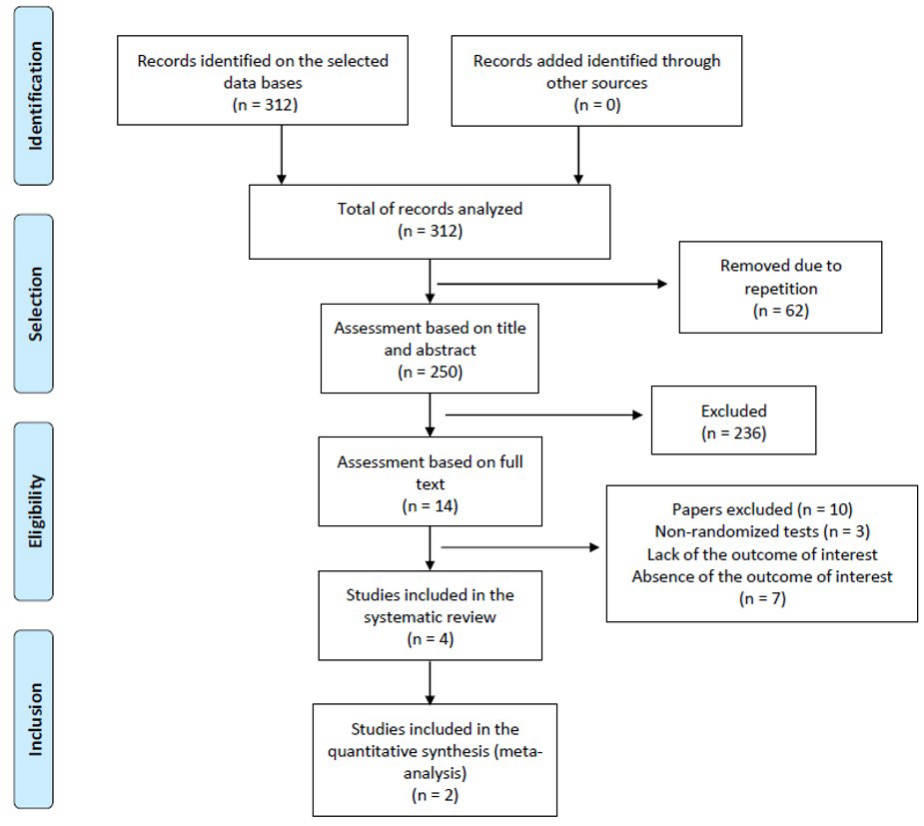
Diagram with the recommendations of the Prisma protocol. Source: Flow Diagram (Prisma 2009)^(14)^

Our search strategy resulted in 312 studies (Figure 2). Four studies remained after the exclusion of repetitions (62), reading of titles, abstracts (236), and full texts (10), meeting all inclusion criteria and considered eligible for the review. Out of these, only two were finally included in the quantitative analysis.

The sample included 165 volunteers (Table 1), predominantly males (85%). There was a loss of tracking of 34% over the period ranging from the randomization start to the end of the follow-up, which occurred from 6 weeks to 24 months^(11,18-20)^.

**Table 1:** Characteristics of the studies included

**AUTHOR/YEAR**	**SAMPLE**	**AGE** (mean)	**ANTINEOPLASTIC TREATMENT**	**RT**	**INTERVENTION PERIOD**	**IG x CG**	**FREQUENCY**	**DURATION**
Carnaby-Mann et al.^(11)^	40	56.5	RT + QT	Conventional (45%)	Throughout the 4 cycles of antineoplastic treatment up to 6 weeks	Exercises x Usual Care	2x/day	45 minutes / session
	(20 IG and 20 CG)			IMRT (55%)				
	82.5%/M							
Messing et al.^(18)^	60	57.5	RT + QT	Conventional* and IMRT*	During the CRT (Except the interval week on the 4^th^ week) and up to 3 months after the CRT	Exercises x Stretching	2x/day	20-30 minutes / session
	(30 IG and 30 CG)						7 days/week	
	90%/M							
Mortensen et al.^(19)^	39	58	RT + QT	Conventional* and IMRT*	Start before the RT up to 11 months	Exercises x Usual Care	3x/day	10-15 minutes / session
	(19 IG and 20 CG)						7 days/week	
	87%/M							
Kotz et al.^(20)^	26	59	RT + QT	Conventional	?	Exercises x Usual Care	3x/day	?
	(13 IG and 13 CG)						7 days/week	
	77%/M							

Caption: IG: intervention group; CG: control group; *no detailed information regarding the number of individuals; ?: No information in the study; RT: radiotherapy; QT: chemotherapy; IMRT: intensity-modulated radiation therapy; CRT: chemotherapy + radiotherapy.

The distribution of the participant's age (57.6 ± 8.2 years old) was close to the usual occurrence of HNC in the age group of 60-75 years^(16)^. This is important information since RT-induced dysphagia tends to be more severe, and sometimes chronic, in elderly individuals^(23)^.

Carnaby-Mann et al.^(11)^ carried out a study with volunteers randomized in three arms, but only the “Pharyngocise” (intervention) and usual care (control) groups were considered in the analysis. Messing et al.^(18)^, Mortensen et al.^(19)^, and Kotz et al.^(20)^ distributed their volunteers into only two arms (intervention and control). Three studies^(11,18,20)^ applied scales for the assessment of oral intake, and two tests^(18,19)^ used videofluoroscopy.

All patients were treated with conventional RT^(11,18-20)^ or intensity-modulated RT (IMRT)^(11,18,19)^. The IMRT is known to preserve the regions close to the tumor, reducing the radiation effects on the stomatognathic functions^(24)^. Radio-induced fibrosis is one of the main undesirable effects of RT and can become chronic in the absence of early intervention. In addition, throughout the RT treatment, the irradiated muscle is modified regarding the distribution of type of fiber, and the predominance is altered to type-I muscle fibers, which lowers the speed of contraction and may slow the swallowing movement and delay the pharyngeal response, which, combined, worsen the risk of aspiration^(25)^.

In addition to the muscle alterations, depending on the irradiated region, RT may promote different degrees of alteration in sensitivity, taste, salivary flow, and laryngeal swelling^(26)^. Either individually or combined, these effects affect the swallowing process leading to significant systemic repercussions that can negatively influence the adherence to the cancer treatment^(27)^, which, in turn, requires the deployment of multidisciplinary prophylactic interventions^(28,29)^.

The intervention protocols of the studies included herein are composed of different techniques of exercises and associated swallowing maneuvers, mostly an adaptation of food consistency. For the volunteers in the control group, the usual care implemented in the patient care routines was preserved by the speech therapy service of the hospital^(11)^: diet supervision and safe nutrition (positioning, volume control, control of pace of supply, instrument adaptation, among others), individualized dietary counseling^(19)^, and reference to a specialized speech therapist to assess the swallowing and treatment of dysphagic symptoms if persisting after the treatment completion^(20)^.

We found no significant change in the nutritional state between the groups by the end of the interventions (Table 2). Such results demonstrate that, in general, dysphagia preventive measures exert no relevant impact on the nutritional state, most likely because, at the early phase of the cancer treatment for HNC, dysphagia is still very incipient for most patients^(30)^. Still, perhaps because at this phase of the treatment, the strong catabolic predominance affects all patients indiscriminately regardless of any stomatognathic alterations that can appear early. In contrast, the finding of equivalent nutritional conditions reveals, indirectly, that nutritional care is extremely relevant for the maintenance of functional capacity.

**Table 2:** Effect of the prophylactic exercises on swallowing

**AUTHOR/YEAR**	**IG PROTOCOL**	**CG PROTOCOL**	**SWALLOWING FUNCTION**	**ORAL INTAKE**	**VAA**	**NUTRITIONAL STATE**
Carnaby-Mann et al.^(11)^	Falsetto, swallowing with effort, tongue counter-resistance, and therabite	Usual Care *	All groups showed a deterioration in the muscle composition	The FOIS score decreased during the treatment in both groups, indicating a greater impairment of the swallowing function	Lower ADV índex in the IG	There was no difference between groups
	+		Three muscles related to the swallowing function showed greater conservation in the IG	The IG showed a higher FOIS median after the treatment, indicating a potential benefit of the intervention		
	Adaptation of diet consistency		The relaxing time was better in the IG than in the CG	The lower degree of dysphagia in the IG, with greater maintenance of the oral nutrition and mouth opening		
			There was no difference between the groups regarding the predominance of aspiration			
Messing et al.^(18)^	Therabite	07/07/2007	The IG presented greater efficacy in swallowing three months after the treatment with less impairment of the PP and TPT	60.6% of the patients needed to change their diet consistency throughout the treatment, with some improvement 6 months after the end of the treatment	There was no significant difference between the groups regarding the use of an alternative route for the diet, mucositis, pain, or weight	
	+	(7 stretching exercises passive of movement amplitude, with 7 repetitions, 7 times a day)				
	Exercises of strength/ oromotor stretching and swallowing maneuvers					
Mortensen et al.^(19)^	Exercises of movement amplitude and resistance	Usual Care *	There was no difference between the groups regarding the swallowing function	?	There was no difference between the groups over the 11 months of tracking	There was no difference between the groups
			Most of the participants (31/39) developed severe dysphagia over the treatment			
Kotz et al.^(20)^	Swallowing with effort, tongue mobility, supraglottic maneuvers, and Mendelsohn	Usual Care *	There was no difference between the groups immediately after the treatment.	There was no difference between the groups (qualitative analysis through the LQQ)	There was no difference between the groups	?
				The IG showed a significant difference in the FOIS in the 3^rd^ and 6^th^ months of follow-up	The average time for the removal of the ADV encompassed 3 months in both groups	

Caption: IG: intervention group; CG: control group; ?: no detailed information; PP: pharyngeal phase; TPT: time of pharyngeal transit; FOIS: functional oral intake scale; ADV: alternative diet via; LQQ: life quality questionnaire.

Even so, the mean difference between the groups by the end of the third month of tracking, assessed through the random effect, reveals that the prophylactic measures significantly increase the FOIS (Functional Oral Intake Scale) score, indicating that the intervention affected the oral intake. The values of mean weighted difference and effect size were 1.27 [IC95%: 0.74 to 1.80] and 3.17 (Cohen D), respectively, in favor of the intervention. However, despite such an expressive effect size, it is worth highlighting that this analysis counted only 33 volunteers in each arm (intervention/control). The low sampling number and the inclusion of only two studies do not invalidate the analysis but imply some caution at the moment of interpreting and transposing the results to daily clinical practice. It is also worth emphasizing the low heterogeneity among the studies (Figure 1); however, even so, the probability of swallowing exercises affecting the progression of dysphagia during RT must be more largely studied.

According to Starmer (2014)^(31)^, evidence suggests that maintaining oral nutrition and practicing swallowing exercises throughout the cancer treatment have a positive impact both on diet consistency and the swallowing physiology, quality of life, and reduction in the use of alternative nutrition routines. Apparently, swallowing exercises reduce the impairment by radio-induced fibrosis, preserving the function of the muscles involved in the stomatognathic functions and contributing to preserving the capacities of mouth opening, chewing, and swallowing food^(31)^.

Atrophy by disuse emerges early and manifests as greater fatigue, and lower strength, in addition to damaged amplitude of movements and motor control^(5,11,12)^. The severity of such an impairment can be closely linked to the early interruption of oral nutrition since the maintenance of this via (or training involving swallowing) provides a lower impact on the muscle involved and increases the possibility of recovery of the muscle homeostasis after the treatment^(5,11)^.

De Felice et al.^(32)^ reinforce the importance of multi-professional actions in the clinical decision-making process to ensure the referring to early patient care. Knowing the complications resulting from the HNC treatment is fundamental to anticipating the intervention of speech therapy since it allows, to some extent, minimizing the harmful effects caused by the antineoplastic treatment on swallowing^(32)^. The data of this meta-analysis reinforce such a recommendation and can contribute to the progress of multi-professional patient care qualification in this clinical scenario^(33)^.

The preventive measures adopted to prevent the progression of dysphagia throughout the HNC treatment impose the patients with a series of indispensable physical and behavioral adaptations to face the clinical condition involved. Therefore, the multi-professional team has an important role when assessing and identifying the symptoms inherent to the treatment by planning and reinforcing the stimuli of adherence to actions aimed at the care entirety^(34,35)^.

Despite the prophylactic intervention has been suggested to benefit swallowing through exercises, it is not possible to state that all patients will preserve or recover their swallowing functionality. It is expected that at least one in 2.15 patients reaches positive results (NNT 2.15) in the effect size analysis.

There was an 81% probability of superiority in the FOIS for the volunteers who practiced the swallowing exercises. In general, the FOIS values for 89% of the volunteers in the intervention group were higher than the mean of the control group. Even so, the limitations of sample size, high loss percentage, discrepancy among the therapeutical programs, and low adherence to the exercises are, according to Lazarus et al.^(36)^, important confounding factors for the analysis of results. In turn, such results cannot be assumed as definitive, but rather partial data given the limitations described.

Apparently, adherent patients have greater chances of achieving benefits that are closer to the superior values of the confidence interval. Thereby, it is reasonable to assume that prophylactic exercises should be encouraged as much as possible since there was no report of undesirable effects or events that could have compromised the RT continuity. These results must be considered with caution, but at the same time, should stimulate further studies. However, we found no evidence indicating any benefit to the patients allocated in the control groups. Thus, the low risk involved in the swallowing exercises and the good probability of benefits justify the prophylactic use of such techniques to manage and control the progression of dysphagia associated with HNC. In addition, other important outcomes should be studied, such as pain (in the orofacial, pharyngeal, and laryngeal regions), and the effectiveness of cough; in addition, other protection maneuvers of lower airways should be included in further studies^(36)^.

Even though this review is focused on the analysis of the prophylactic effect of swallowing exercise protocols on the progression of dysphagia, it is worth highlighting the valuable contribution of other associated techniques. Laser therapy, for example, is indicated to prevent or treat mucositis and can improve the swallowing pattern by reducing odynophagia during nutrition^(37)^. Likewise, there is some evidence that electrostimulation, in association with exercises, favors the maintenance of muscle function, conservation, and/or recovery of the salivary flow, in addition to reducing laryngeal swelling^(38-40)^.

It is still not possible to determine the ideal moment to start the prophylactic intervention or the most efficient therapeutic strategies. Further studies should clarify issues concerning the number of sessions, weekly frequency, intervention duration, types of exercises, muscle overload intensity, number of repetitions/series, and other components that constitute a complete recovery program. So far, it is known that a certain benefit is provided, which justifies further efforts to enlarge and deepen the evidence.

The main risks of bias assessed based on the Pedro scale^(17)^ refer to the blinding of the evaluators, blinding of the participants, and absence of information concerning the protocols used in the clinical practice (Chart 1). However, the blinding of this type of intervention (active swallowing exercises) is certainly very unlikely, especially in the context of such different therapeutic proposals (usual care versus therapeutic exercises). The low number of studies and sampling limited the analyses of the effect size, invalidating the study of subgroups and correlation. Combined with such limitations, the strength of the evidence is weakened.

The quality of evidence (based on the GRADE system) summarizes the evaluations performed for the body of evidence present in the meta-analysis and the narrative description of the systematic review. The quality of evidence was evaluated as low due to the risk of bias in the individual studies and the issues related to the imprecision of results. Chart 2 presents the justifications for each evaluation in detail.

**Chart 2:** List of findings of the studies included in this review systematic

Population: patients com cancer of the head and neck						
Context: outpatient						
Intervention: prophylactic swallowing exercises						
Comparison: usual care						
**Outcomes**	**Nº of participants analyzed (Nº of studies)**	**Studies included**	**Statistical heterogeneity (*I^2^* )**	**Effect size (IC95%)**	**Quality of evidence (GRADE)**	**Interpretation**
**Functional Oral Intake Scale (FOIS)** ^a^	33 (2)	^(11,20)^	0%	MWD	⨁⨁◯◯ **LOW** ^c,d^	Evidence of low quality suggests that prophylactic swallowing exercises decrease the progression of dysphagia according to the FOIS
				1.27 (0.74 to 1.80)		
**Progression of Dysphagia assessed based on the FOIS** ^b^	165 (4)	^(11,18-20)^	NA	Two studies demonstrate benefits and two absence of effect		

a Assessment of the quality of evidence only for the studies included in the meta-analysis^(11,20)^;

b Assessment of the quality of evidence for all studies included in the review systematic^(11,18-20)^ in narrative synthesis, according to recommendations^(41)^;

The GRADE approach to assessing the quality of evidence;

c Scaled to low on a risk level of bias in the individual studies due to the absence of masking for a subjective outcome evaluation;

d Scaled to low on an imprecision level due to the large IC95%. Two studies reported favorable results of the intervention, while the other two reported nullity, mostly likely the low statistical power due to the small number of participants analyzed. In addition, the total number of patients included in all four studies was below 200, generating doubts regarding the reliability of the results in terms of statistical accuracy considering the Optimum Size of the Information^(42)^

Caption: NA: Not Applicable. Means represent the post-treatment value of each group; MWD = represents the difference between the groups in the post-treatment means; IC95%, = confidence interval of 95%; FOIS: functional oral intake scale.

## CONCLUSION

Based on the evidence presented, it is reasonable to assume that patients with HNC can experience some positive effects on oral intake through prophylactic swallowing exercises compared with those who are not subjected to this therapeutic measure throughout radiotherapy. However, the low quality of evidence and the limited details on the actions implemented in the patient care protocols justify further studies.

## Supplementary Material

Supplementary material accompanies this paper.

Caption S1 Preventive measures for the progression of dysphagia in patients with cancer of head and neck subjected to radiotherapy: A systematic review with meta-analysis.

This material is available as part of the online article from: https://www.scielo.br/j/codas
