# Supplementary material for: Preventive measures for the progression of dysphagia in patients with cancer of head and neck subjected to radiotherapy: a systematic review with meta-analysis
Source: Codas. 2023 May 1;35(2):e20210246. doi: 10.1590/2317-1782/20232021246en (PMC10162649; doi:10.1590/2317-1782/20232021246en)
Supplement: Legenda S1 [file codas-35-2-e20210246-supl.pdf]

MATERIAL SUPLEMENTAR

BUSCA PUBMED

(((Head and Neck Neoplasms) AND (((Deglutition Disorders) OR (Dysphagia)) OR (Swallowing Disorders))) AND ((randomized controlled trial[Publication Type] OR (randomized[Title/Abstract] AND controlled[Title/Abstract] AND trial[Title/Abstract]))) AND ((Prevention) OR (Prophylactic))

75 artigos

BUSCA SCOPUS - Advanced

(ALL (head AND neck AND neoplasms) AND ALL (deglutition AND disorders) OR ALL (dysphagia) OR ALL (swallowing AND disorders) AND TITLE-ABS-KEY (randomized AND controlled AND trial) OR TITLE-ABS-KEY (randomized) AND TITLE-ABS-KEY (controlled) AND TITLE-ABS-KEY (trial) AND TITLE-ABS-KEY (prevention) OR TITLE-ABS-KEY (prophylactic))

64 artigos

BUSCA EMBASE

('head and neck neoplasms' AND 'deglutition disorders' OR dysphagia OR 'swallowing disorders') AND (prevention OR prophylactic) AND ('randomized controlled trial'/mj OR randomized:ti,ab) AND controlled:ti,ab AND trial:ti,ab

114 artigos

BUSCA BVS

(tw:(Head and Neck Neoplasms)) AND (tw:(Deglutition Disorders)) OR (tw:(Dysphagia)) OR (tw:(Swallowing Disorders)) AND (tw:(Randomized Controlled Trial)) OR (tw:(Randomized)) AND (tw:(controlled)) AND (tw:(trial)) AND (tw:(Prevention)) OR (tw:(Prophylactic))

59 artigos

MATERIAL SUPLEMENTAR

BUSCA PUBMED

(((Head and Neck Neoplasms) AND (((Deglutition Disorders) OR (Dysphagia)) OR (Swallowing Disorders))) AND ((randomized controlled trial[Publication Type] OR (randomized[Title/Abstract] AND controlled[Title/Abstract] AND trial[Title/Abstract])))) AND ((Prevention) OR (Prophylactic))

75 artigos

BUSCA SCOPUS - Advanced

(ALL (head AND neck AND neoplasms) AND ALL (deglutition AND disorders) OR ALL (dysphagia) OR ALL (swallowing AND disorders) AND TITLE-ABS-KEY (randomized AND controlled AND trial) OR TITLE-ABS-KEY (randomized) AND TITLE-ABS-KEY (controlled) AND TITLE-ABS-KEY (trial) AND TITLE-ABS-KEY (prevention) OR TITLE-ABS-KEY (prophylactic))

64 artigos

BUSCA EMBASE

('head and neck neoplasms' AND 'deglutition disorders' OR dysphagia OR 'swallowing disorders') AND (prevention OR prophylactic) AND ('randomized controlled trial'/mj OR randomized:ti,ab) AND controlled:ti,ab AND trial:ti,ab

114 artigos

BUSCA BVS

(tw:(Head and Neck Neoplasms)) AND (tw:(Deglutition Disorders)) OR (tw:(Dysphagia)) OR (tw:(Swallowing Disorders)) AND (tw:(Randomized Controlled Trial)) OR (tw:(Randomized)) AND (tw:(controlled)) AND (tw:(trial)) AND (tw:(Prevention)) OR (tw:(Prophylactic))

59 artigos
