# Supplementary material for: Preventive measures for the progression of dysphagia in patients with cancer of head and neck subjected to radiotherapy: a systematic review with meta-analysis
Source: Codas. 2023 May 1;35(2):e20210246. doi: 10.1590/2317-1782/20232021246en (PMC10162649; doi:10.1590/2317-1782/20232021246en)
Supplement: Translation

# Translation: Medidas de prevenção da progressão da disfagia em pacientes com câncer de cabeça e pescoço submetidos a radioterapia: uma revisão sistemática com meta-análise

**DOI:** 10.1590/2317-1782/20232021246pt

**Authors:** Amanda Guterres Beuren, Émille Dalbem Paim, Nathália da Silva Flores, Vera Beatris Martins, Fabricio Edler Macagnan

**Affiliations:** 1 Universidade Federal de Ciências da Saúde de Porto Alegre - UFCSPA - Porto Alegre (RS), Brasil.; 2 Irmandade Santa Casa de Misericórdia de Porto Alegre - ISCMPA - Porto Alegre (RS), Brasil.; 3 Departamento de Fisioterapia, Universidade Federal de Ciências da Saúde de Porto Alegre - UFCSPA - Porto Alegre (RS), Brasil.

**Keywords:** Profilaxia, Medidas Preventivas, Disfagia, Neoplasias de Cabeça e Pescoço, Radioterapia

## Abstract

**Objetivo:**

Identificar os efeitos de medidas profiláticas, não farmacológicas, sobre a progressão da disfagia em pacientes com câncer de cabeça e pescoço submetidos a radioterapia.

**Estratégia de pesquisa:**

A busca foi realizada nas bases de dados Medline (via PubMed), Scopus e Embase, assim como na literatura cinzenta.

**Critérios de seleção:**

Foram incluídos ensaios clínicos randomizados, com pacientes adultos (≥ 18 anos) e diagnóstico de câncer de cabeça e pescoço, tratados com radioterapia (associada ou não à cirurgia e quimioterapia) submetidos a protocolos não farmacológicos de prevenção da disfagia.

**Análise dos dados:**

O risco de viés foi avaliado por meio da escala PEDRO e a qualidade global da evidência foi avaliada de acordo com o instrumento GRADE.

**Resultados:**

Foram considerados elegíveis 4 estudos, e desses, dois foram incluídos na metanálise. O resultado favoreceu o grupo intervenção, com diferença média de 1,27 [IC 95%: 0,74 à 1,80]. Houve baixa heterogeneidade e a pontuação média para risco de viés foi de 7,5 de um total de 11 pontos. A falta de detalhamento nos cuidados com os vieses de seleção, performance, detecção, atrito e de relato contribuíram para o julgamento da qualidade da evidência, considerada baixa.

**Conclusão:**

Medidas profiláticas de contenção da disfagia podem promover importantes benefícios sobre a ingesta oral dos pacientes com câncer de cabeça e pescoço, quando comparados aqueles que não realizaram tal medida terapêutica ao longo da radioterapia.

## INTRODUÇÃO

A disfagia é uma alteração comum nos pacientes com câncer de cabeça e pescoço (CCP)^(1)^. Esta disfunção causa limitação na alimentação por via oral e prejuízo no estado nutricional aumentando o risco de pneumonias aspirativas recorrentes^(2)^. Devido ao papel que a alimentação por via oral representa na funcionalidade e qualidade de vida dos indivíduos, é fundamental detectar precocemente as alterações que sinalizam o início da disfagia, visando minimizar o impacto que este distúrbio promove no curso do tratamento oncológico^(3)^.

Sabe-se que pacientes com CCP submetidos à radioterapia (RT) podem apresentar maior prejuízo na deglutição quando comparados àqueles que realizam apenas intervenção cirúrgica. Os efeitos adversos provocados pela radiação como mucosite, xerostomia, dor, reações cutâneas e edema, aliados à fibrose tecidual, contribuem para o declínio na deglutição^(4)^.

De um modo geral toda a musculatura esquelética é afetada pelo precário estado nutricional promovido pela redução da capacidade de engolir, e nesse processo a própria musculatura envolvida no processo de deglutição perde performance agravando o quadro clínico^(5,6)^. É comum, nestes casos, a utilização de vias alternativas para alimentação durante o tratamento, no entanto sabe-se que, embora necessária, a alimentação artificial pode ser insuficiente para manter o estado nutricional e pode ainda agir negativamente sobre a evolução da disfagia^(7-9)^.

Devido aos efeitos adversos que iniciam durante o tratamento, os indivíduos podem apresentar limitação ou interrupção da ingesta de alimentos por via oral e esse desuso da musculatura envolvida na deglutição pode estimular a remodelação dos músculos e possivelmente exacerbar a fibrose e o edema radioinduzido^(5,6)^. Os músculos esqueléticos já começam a mostrar evidências de atrofia por desuso apenas algumas horas após a imobilização^(5,10)^.

A reabilitação da disfagia associada ao CCP, envolve múltiplas abordagens principalmente nos pacientes tratados com radioterapia, em função dos efeitos de longo prazo induzidos pela radiação ionizante (fibrose tecidual). Nos últimos anos, tem-se investigado o efeito de diferentes modelos de profilaxia da disfagia ao longo do tratamento antineoplásico com o objetivo de identificar o momento ideal para iniciar a intervenção^(9,11)^.

Tais medidas são importantes pois a RT, apesar dos efeitos antitumorais desejados, promove efeitos cumulativos em vias moleculares da musculatura esquelética o que implica em mudança na configuração muscular com mutação do tipo e tamanho das fibras musculares, aumento de tecido adiposo local e redistribuição de fibras dentro do músculo^(5,6)^.

Embora essas medidas preventivas estejam sendo investigadas e por vezes utilizadas, ainda não existe um consenso quanto ao efeito de medidas profiláticas sobre o grau da disfagia. Nesse sentido, este estudo de revisão sistemática da literatura visou avaliar o efeito de intervenções profiláticas na progressão da disfagia associada ao CCP de pacientes submetidos à radioterapia no intuito de direcionar e auxiliar a tomada de decisão no manejo clínico precoce^(12)^.

## OBJETIVO

O objetivo deste estudo foi identificar o efeito de medidas profiláticas, não farmacológicas, sobre a progressão da disfagia de pacientes com câncer de cabeça e pescoço submetidos à radioterapia.

## ESTRATÉGIA DE PESQUISA

O estudo está baseado nas recomendações preconizadas pelo *Cochrane Handbook* ^(13)^. A revisão foi descrita de acordo com o *Preferred Reporting Items for Systematic Reviews and Meta-Analyses* (PRISMA Statement) conforme checklist que consta no Material Suplementar^(14)^. Posteriormente, foi registrada no *International Prospective Register of Systematic Reviews* (PROSPERO), com a seguinte identificação: CRD42021226726 e utilizou-se a estratégia PICO (Population, Intervention, Comparison/control, Outcome)^(15,16)^.

## CRITÉRIOS DE SELEÇÃO

Foram incluídos apenas ensaios clínicos randomizados que analisaram em um braço do estudo o efeito profilático de intervenções não farmacológicas sobre a disfagia comparando os resultados a um grupo controle. A amostra se deu por pacientes adultos com diagnóstico de CCP e indicação de radioterapia, associados ou não a quimioterapia e cirurgia, com ou sem disfagia no início do estudo.

Foi considerado desfecho primário: progressão do grau da disfagia, avaliada pela diferença entre a avaliação inicial e final; não houveram restrições quanto ao método ou instrumento de mensuração da disfagia, pois os resultados foram normalizados através da diferença entre a graduação inicial e final do estado de disfagia. Como desfecho secundário considerou-se a análise do perfil nutricional e presença de via alternativa para alimentação.

Quanto a intervenção de interesse, não houve restrição ou direcionamento para uma ou outra medida profilática de prevenção ou progressão (agravo) da disfagia. Não houve comparação entre técnicas de profilaxia específicas, apenas a medida do efeito em relação aos cuidados usuais (normalmente realizadas através de orientações) ou em relação à tratamento placebo ou sham.

Foram excluídos estudos que não envolvessem intervenção não farmacológica para prevenção da disfagia e/ou que não envolvessem avaliação fonoaudiológica.

A busca foi realizada nas principais bases de dados: Medline (via PubMed), Scopus e Embase, assim como na literatura cinzenta por meio do Clinical Trial, WHO International Clinical Trials Registry Platform, REBEC OpenGrey e resumos de congressos potencialmente relevantes nos últimos 5 anos. A busca manual por artigos também foi realizada por uma triagem das referências dos artigos incluídos na presente revisão sistemática. O processo da seleção dos estudos iniciou logo após a última busca (dezembro de 2020)

Não houve restrição de idiomas ou data de publicação dos estudos das bases de dados e da literatura cinzenta. Foram utilizadas diversas variações morfológicas e termos sinônimos relacionados às seguintes palavras: “*Head and neck neoplasms*”, “*Deglutition disorders*”, “*Dysphagia*”, “*Swallowing disorders*”, “*Prevention*”, “*Prophylactic*”, “*Randomized controlled trial*”, “*randomized*”, “*controlled*” e “*trial*”. A estratégia de busca completa para cada base bibliográfica está descrita no Material Suplementar.

Após exclusão de artigos em duplicata, dois revisores (AGB e NSF) avaliaram de forma independente os títulos e resumos obtidos. Os artigos foram selecionados com base nos critérios de elegibilidade em um software de gerenciamento bibliográfico (Mendeley). Nessa etapa, as discordâncias foram analisadas por um terceiro revisor (FEM).

Após a exclusão realizada com base nos títulos e resumos, os textos completos foram lidos, pelos dois revisores, para a decisão final de inclusão ou exclusão, resolvendo qualquer discordância por intermediação do terceiro revisor (FEM).

A extração de dados foi realizada por dois autores (AGB e NSF), através de um formulário padronizado com as seguintes informações: delineamento do estudo, primeiro autor, ano de publicação, local onde o estudo foi conduzido, tamanho amostral, características clínicas dos voluntários, descrição detalhada das intervenções implementadas no grupo intervenção e controle, e os valores pré e pós-tratamento para os resultados obtidos nas diferentes escalas de avaliação da disfagia, tanto para grupo controle quanto para grupo intervenção.

Todas as informações foram organizadas e armazenadas no software Excel, e as discordâncias entre os autores foram resolvidas por consenso com o terceiro revisor, que realizou a checagem de dados.

## ANÁLISE DOS DADOS

Dois revisores independentes (EDP e VBM) avaliaram o risco de viés (escala PEDro)^(17)^, sendo que os julgamentos finais foram discutidos e definidos em conjunto com todos os autores. Para pontuar nos critérios da escala, as informações deveriam ser claras e objetivas, caso contrário a pontuação seria considerada nula, os resultados estão expressos no Quadro 1.

**Quadro 1:** Avaliação da qualidade metodológica dos artigos conforme a escala Pedro^17^

CLASSIFICAÇÃO PEDRo/ ARTIGOS		Carnaby-Mann et al.^(11)^	Messing et al.^(18)^	Mortensen et al.^(19)^	Kotz et al.^(20)^
Validade Externa	1 Critérios de inclusão	S	S	S	S
(Max = 1)					
Validade Interna	2 Alocação aleatória	S	S	S	S
(Max=8)	3 Alocação secreta	N	S	N	N
	4 Grupo semelhante no início do estudo	S	S	S	S
	5 Cegamento dos participantes	N	N	N	N
	6 Cegamento dos terapeutas	N	N	N	N
	7 Cegamento da avaliação	N	S	N	N
	8 Análise de 86% da amostra	S	S	S	S
	9 Análise de intenção de tratamento	S	S	S	S
Interpretação dos desfechos	10 Comparação entre os grupos	S	S	S	S
(Max=2)	11 Medidas de tendência central e de dispersão	S	S	S	S
Total de pontos	-	7	9	7	7
(Max = 11)					

Legenda: S = sim; N = não; Max = máximo

A qualidade global da evidência foi avaliada com base na abordagem GRADE^(21,22)^. Para cada desfecho, a qualidade da evidência é inicialmente considerada ‘alta’ e subsequentemente pode ser graduada para baixo aos níveis ‘moderada’, ‘baixa’ ou ‘muito baixa’ qualidade, a depender da avaliação de cinco critérios: risco de viés dos estudos individuais, evidência indireta, heterogeneidade, imprecisão e risco de viés de publicação. A qualidade da evidência foi separadamente julgada de duas formas: a) para o corpo de evidência composto apenas pelos estudos incluídos na metanálise; e b) para o corpo de evidência incluído na presente revisão sistemática, composto pela totalidade de estudos individuais narrativamente sintetizados^(22)^.

Para avaliação do viés de publicação foi utilizada regressão linear das estimativas do efeito da intervenção por sua variância inversa por meio do Egger test e do gráfico Funnel Plot.

Conforme descrito na Figura 1, apenas os resultados de dois estudos^(11,20)^ foram estatisticamente compilados em uma meta-análise. A análise foi conduzida utilizando o método do inverso das variâncias e estimador de Der Simonian *and* Laird para τ^2^ em modelo de efeitos randômicos, o que permite incorporar estatisticamente a variabilidade entre estudos na estimativa de efeito final. Para desfechos contínuos, foram utilizados os dados das médias pós-tratamento de cada grupo para cálculo do tamanho de efeito (Cohen D) a partir da diferença média ponderada (DMP).

**Figura 1:** Metanálise do efeito profilático sobre a progressão a disfagia de pacientes com câncer de cabeça e pescoço submetidos ao tratamento antineoplásico. A graduação da disfagia foi avaliada através da escala FOIS em ambos estudos com período de comparação semelhante, sendo 6 semanas (10) e 3 meses (20) com o objetivo de redução do gap entre os estudos

Para inclusão de resultados dos estudos que não relataram os dados em média e desvio padrão (DP) nas meta-análises, a conversão de dados de mediana para média foi realizada pelo método de Hozo^(15)^. Todas as análises foram conduzidas no software RStudio (versão 1.3.1093) com o pacote ‘meta’ na linguagem R (versão 4.0.3).

A heterogeneidade estatística foi avaliada quantitativamente utilizando a estatística *I^2^* e o teste de *χ^2^* . A interpretação da heterogeneidade estatística seguiu as orientações mais recentes (Cochrane Handbook, versão 6.0)^(13)^, onde *I^2^* até 40% representa heterogeneidade de efeito trivial de 30 a 60% representa heterogeneidade moderada; de 50 a 90% representa heterogeneidade substancial e de 75% a 100% apresenta heterogeneidade considerável.

## RESULTADOS

A estratégia de busca resultou em 312 estudos (Figura 2), que após exclusão de duplicatas (62), leitura de título e resumo (236) e textos completos (10), resultou em 4 estudos que apresentaram todos os critérios de inclusão e foram considerados elegíveis para a revisão. Destes, apenas dois foram incluídos na análise quantitativa.

**Figura 2:** Diagrama de acordo com as recomendações do protocolo Prisma. Fonte: Flow Diagram (Prisma 2009)^(14)^

A amostra incluiu 165 voluntários (Tabela 1), sendo que o sexo masculino foi o mais prevalente (85%). Houve perda de seguimento de 34% no período compreendido entre o início da randomização até o final do acompanhamento que foi de 6 semanas a 24 meses^(11,18-20)^.

**Tabela 1:** Características dos estudos incluídos

**AUTOR/ANO**	**AMOSTRA**	**IDADE**	**TRATAMENTO**	**RT**	**PERÍODO DA INTERVENÇÃO**	**GI x GC**	**FREQUÊNCIA**	**DURAÇÃO**
		(média)	**ANTINEOPLÁSICO**					
Carnaby-Mann et al.^(11)^	40	56,5	RT + QT	Convencional (45%)	Ao longo de 4 ciclos do tratamento antineoplásico até um máximo de 6 semanas	Exercícios x Cuidados habituais	2x/dia	45 minutos / sessão
	(20 GI e 20 GC)			IMRT (55%)				
	82,5%/H							
Messing et al.^(18)^	60	57,5	RT + QT	Convencional* e IMRT*	Durante a QT+RT (Exceto semana de intervalo na semana 4) e até 3 meses após a QT+RT	Exercícios x Alongamentos	2x/dia	20-30 minutos / sessão
	(30 GI e 30 GC)						7 dias/semana	
	90%/H							
	
Mortensen et al.^(19)^	39	58	RT + QT	Convencional* e IMRT^*^	Início antes da RT, até 11 meses	Exercícios x Cuidados habituais	3x/dia	10-15 minutos / sessão
	(19 GI e 20 GC)						7 dias/semana	
	87%/H							
Kotz et al.^(20)^	26	59	RT + QT	Convencional	?	Exercícios x Cuidados habituais	3x/dia	?
	(13 GI e 13 GC)						7 dias/semana	
	77%/H							

Legenda: GI: grupo intervenção; GC: grupo controle; H: homens; RT: radioterapia; QT: quimioterapia; *sem informação detalhada quanto ao número de indivíduos; ?: Não constava a informação no estudo; RT: radioterapia; QT: quimioterapia; IMRT: radioterapia de intensidade modulada.

A distribuição da idade dos participantes (57,6 ± 8,2 anos) ficou próxima da incidência de CCP que comumente ocorre na faixa etária de 60-75 anos^(16)^. Esse é um dado importante, pois a disfagia induzida pela RT tende a ser mais grave, e por vezes crônica, em indivíduos idosos^(23)^.

No estudo realizado por Carnaby-Mann et al.^(11)^ os voluntários foram randomizados em três braços, mas apenas os grupos “Pharyngocise” (intervenção) e de cuidados usuais (controle) foram considerados na análise. Nos estudos de Messing et al.^(18)^, Mortensen et al.^(19)^ e Kotz et al.^(20)^, a distribuição dos voluntários foi realizada em apenas dois braços (intervenção e controle). Três estudos^(11,18,20)^ aplicaram escalas para avaliação da ingestão oral e dois ensaios^(18,19)^ utilizaram a videofluoroscopia.

Todos os pacientes foram tratados com RT convencional^(11,18-20)^ ou RT de intensidade modulada (IMRT)^(11,18,19)^. Sabe-se que a IMRT preserva as regiões próximas ao tumor, reduzindo os efeitos da radiação sobre as funções estomatognáticas^(24)^. A fibrose radioinduzida é um dos principais efeitos indesejáveis da RT, e pode tornar-se crônica na ausência de intervenção precoce. Além disso, ao longo do tratamento com RT, a musculatura irradiada sofre modificação na distribuição do tipo de fibra alterando o predomínio para fibras musculares do tipo I, o que reduz a velocidade de contração podendo gerar lentidão do movimento de deglutição e atraso da resposta faríngea, que em conjunto agravam o risco de aspiração^(25)^.

Adicionalmente às alterações musculares, a RT, dependendo da região irradiada, pode promover diferentes graus de alteração da sensibilidade, paladar, fluxo salivar e edema laríngeo^(26)^. Separadamente ou em conjunto estes efeitos afetam o processo de deglutição levando à repercussões sistêmicas significativas, capazes de influenciar negativamente na adesão ao tratamento oncológico^(27)^, o quê, por sua vez, torna necessário a implementação de intervenções multidisciplinares profiláticas^(28,29)^.

Os protocolos de intervenção, dos estudos incluídos nesta revisão, foram compostos por diferentes técnicas de exercícios e manobras de deglutição associadas, em sua maioria, a adaptação de consistência alimentar. Para os voluntários do grupo controle, foram mantidos os cuidados usuais implementados nas rotinas assistenciais como: supervisão para alimentação e cuidados para alimentação segura (posicionamento, controle de volume, controle de ritmo de oferta, adaptação de instrumento, dentre outros) pelo serviço de fonoaudiologia do hospital^(11)^, aconselhamento dietético individualizado^(19)^ e encaminhamento a um fonoaudiólogo especializado para avaliação da deglutição e tratamento de sintomas disfágicos, se estivessem presentes após a conclusão do tratamento^(20)^.

Não foi identificada mudança significativa no estado nutricional entre os grupos ao final das intervenções (Tabela 2). Estes resultados demonstram que, de um modo geral, as medidas de prevenção da disfagia não exercem impacto relevante sobre o estado nutricional, provavelmente porque, na fase precoce do tratamento oncológico para CCP a disfagia é ainda muito incipiente para a maioria dos pacientes^(30)^ ou talvez porque nessa fase do tratamento, o forte predomínio catabólico afete indiscriminadamente todos os pacientes a despeito de quaisquer alterações estomatognáticas que precocemente possam ocorrer. Por outro lado, a constatação de condições nutricionais equivalentes revela, de forma indireta, a importância dos cuidados nutricionais tão importantes para a manutenção da capacidade funcional.

**Tabela 2:** Efeito dos exercícios profiláticos na deglutição

**AUTOR/ANO**	**PROTOCOLO GI**	**PROTOCOLO GC**	**FUNÇÃO DE DEGLUTIÇÃO**	**INGESTA ORAL**	**VAA**	**ESTADO NUTRICIONAL**
Carnaby-Mann et al.^(11)^	Falsete, deglutição com esforço, contra-resistência de língua e Therabite	Cuidados usuais*	Todos os grupos demonstraram deterioração na composição muscular	Houve redução no escore FOIS durante o tratamento em ambos os grupos, indicando maior comprometimento da função de deglutição	Menor índice de VAA no GI	Não houve diferença entre os grupos
	+		Três músculos relacionadas à função de deglutição demonstraram maior preservação no GI	O GI demonstrou uma mediana de FOIS maior após o tratamento indicando possível benefício da intervenção		
	Adaptação de consistências alimentares		O tempo de relaxamento foi melhor no GI quando comparado ao GC	Menor grau de disfagia no GI, com maior manutenção da dieta via oral e abertura de boca		
			Não houve diferença entre os grupos quanto à prevalência de aspiração			
Messing et al.^(18)^	Therabite	07/07/2007	O GI apresentou maior eficácia na deglutição 3 meses após o tratamento com menor comprometimento da FF e TTF	60.6% dos pacientes necessitaram de modificação na consistência da dieta durante o tratamento, com melhora após 6 meses de término do tratamento	Não houve diferença significativa entre os grupos quanto ao uso de via alternativa para alimentação, mucosite, dor ou peso	
	+	(7 alongamentos passivos da amplitude de movimento, realizados 7 repetições, 7 vezes ao dia)				
	Exercícios de força/alongamento oromotor e manobras de deglutição					
Mortensen et al.^(19)^	Exercícios de amplitude de movimento e exercícios de resistência	Cuidados usuais*	Não houve diferença entre os grupos quanto à função de deglutição.	?	Não houve diferença entre os grupos durante os 11 meses de seguimento	Não houve diferença entre os grupos
			A maioria dos participantes (31/39) desenvolveu disfagia grave durante o tratamento			
Kotz et al.^(20)^	Deglutição com esforço, mobilidade de língua, manobras supra-glótica e Mendelsohn	Cuidados usuais*	Não houve diferença entre os grupos imediatamente após o tratamento.	Não houve diferença entre os grupos (análise qualitativa por meio de QQV)	Não houve diferença entre os grupos	?
				O GI demonstrou diferença significativa na FOIS no 3º e 6º mês de follow-up.	O tempo médio para retirada da VAA foi de 3 meses, em ambos grupos.	

Legenda: GI: grupo intervenção; GC: grupo controle; ?: sem informação detalhada; FF: fase faríngea; TTF: tempo de trânsito faríngeo; FOIS: escala funcional de ingestão por via oral; VAA: via alternativa para alimentação; QQV: questionário de qualidade de vida.

Ainda assim, a média da diferença entre os grupos ao final do terceiro mês de seguimento, avaliada através do efeito randômico, mostra que as medidas profiláticas aumentam significativamente a pontuação na escala FOIS (Functional Oral Intake Scale) o que significa dizer que houve efeito da intervenção sobre a ingesta oral. A média da diferença ponderada foi de 1,27 [IC95%: 0,74, à 1,80] e o tamanho do efeito foi de 3,17 (Cohen D) à favor da intervenção. Contudo, embora o tamanho do efeito tenha sido expressivo, cabe salientar que esta análise contou com apenas 33 voluntários em cada braço (intervenção/controle). O baixo número amostral e a inclusão de apenas dois estudos não inviabilizam a análise, mas requer cautela no momento de interpretar e transpor os resultados para a prática clínica diária. É importante ressaltar também que houve baixa heterogeneidade entre os estudos (Figura 1), mas ainda assim a investigação da probabilidade dos exercícios de deglutição apresentarem efeito sobre a progressão da disfagia durante a RT precisa ser ampliada. Segundo a revisão de Starmer (2014)^(31)^, evidências sugerem que manter a dieta por via oral e praticar exercícios de deglutição durante o tratamento oncológico têm impacto positivo tanto sobre a consistência da dieta quanto na fisiologia da deglutição, qualidade de vida e redução do uso de vias alternativas de alimentação. Aparentemente, os exercícios de deglutição reduzem o comprometimento por fibrose radioinduzida, preservando a função dos músculos envolvidos nas funções estomatognáticas e contribuindo para a manutenção da capacidade de abertura da boca, mastigação e deglutição dos alimentos^(31)^.

A atrofia por desuso, que surge precocemente, se manifesta com aumento da fadiga, redução da força, prejuízo na amplitude dos movimentos e no controle motor^(5,11,12)^. A gravidade do comprometimento pode estar intimamente ligada a interrupção precoce da alimentação por via oral, pois com a manutenção desta via (ou treinamento envolvendo a deglutição) menor será o impacto na musculatura envolvida e maior será a possibilidade de recuperação da homeostase muscular após o tratamento^(5,11)^.

De Felice et al.^(32)^ reforçam a importância da atuação multiprofissional na tomada de decisão clínica que garanta o direcionamento assistencial precoce. Conhecer as complicações decorrentes do tratamento do CCP é fundamental para a antecipação da intervenção fonoaudiológica, pois é possível, em certa medida, minimizar os efeitos deletérios que o tratamento antineoplásico promove sobre a deglutição^(32)^. Os dados desta metanálise reforçam essa recomendação e podem contribuir para o progresso da qualificação assistencial multiprofissional neste cenário clínico^(33)^.

As medidas preventivas adotadas para conter a progressão da disfagia ao longo do tratamento do CCP, exigem dos pacientes uma série de adaptações físicas e comportamentais imprescindíveis para o enfrentamento que esta condição clínica impõe. Nesse sentido, a equipe multiprofissional assume papel importante, avaliando e identificando os sintomas inerentes ao tratamento, para planejar e reforçar os estímulos de adesão às condutas pertinentes visando a integralidade do cuidado^(34,35)^.

Embora tenha sido identificado benefício na intervenção profilática para a deglutição por meio de exercícios, não se pode afirmar que todos os pacientes irão manter ou recuperar a funcionalidade da deglutição. Na análise do tamanho do efeito espera-se que um paciente em cada 2.15 possa ter resultados positivos (NNT 2.15).

A probabilidade de superioridade na escala FOIS foi de 81% para os voluntários que praticaram os exercícios de deglutição. De um modo geral 89% dos voluntários do grupo intervenção apresentaram valores na escala FOIS superiores à média do grupo controle. Ainda assim, em função das limitações de tamanho amostra, elevado percentual de perda, discrepância considerada entre programas terapêuticos e baixa adesão aos exercícios instituídos que, segundo Lazarus, et al. (2014)^(36)^, é um importante fator confundidor para a análise dos resultados, esses resultados não podem ser assumidos como definitivos e sim como dados parciais dadas as limitações descritas.

Ao que tudo indica, pacientes aderentes têm mais chances de alcançar benefícios próximos aos valores superiores do intervalo de confiança. Sendo assim, é razoável assumir que os exercícios profiláticos devem ser encorajados ao máximo, pois não houve relato de efeitos indesejados ou eventos que tenham comprometido a continuidade da RT. Esses resultados devem ser considerados com cautela, mas ao mesmo tempo, devem servir de estímulo para novos estudos. No entanto, não encontramos indícios que sinalizem qualquer benefício aos pacientes alocados nos grupos controles. Sendo assim, o baixo risco envolvido com o emprego dos exercícios de deglutição e a boa probabilidade de benefícios justificam o uso profilático destas técnicas para o manejo e controle da progressão da disfagia associada ao CCP. Além disso, outros desfechos importantes poderiam ser estudados, como por exemplo dor (na região orofacial, faríngea e laríngea), efetividade da tosse e demais manobras de proteção de vias aéreas inferiores poderiam ser incluídos em estudos futuros^(36)^.

Embora essa revisão tenha centralizado a análise no efeito profilático de protocolos de exercícios de deglutição sobre a progressão da disfagia, é válido ressaltar a valiosa contribuição que outras técnicas podem gerar quando associadas. A laserterapia, por exemplo, é indicada para a prevenção ou para o tratamento da mucosite e pode melhorar o padrão de deglutição, visto que reduz a odinofagia durante a alimentação^(37)^. Do mesmo modo, existem evidências de que a eletroestimulação, se utilizada associada aos exercícios, auxilia na manutenção da função muscular, preservação e/ou recuperação do fluxo salivar e redução do edema laríngeo^(38-40)^.

Ainda não é possível afirmar qual o momento ideal para iniciar a intervenção profilática, nem quais as estratégias terapêuticas são mais eficazes. Mais estudos deverão elucidar questões referentes ao número de sessões, frequência semanal, duração da intervenção, tipos de exercícios, intensidade de sobrecarga muscular, número de repetições/séries e demais componentes que constituem um programa completo de reabilitação. Por ora sabemos que existe certo benefício e isso justifica empreender mais esforço na ampliação e aprofundamento das evidências.

Os principais riscos de viés, avaliados na escala Pedro^(17)^, referem-se ao cegamento dos avaliadores, cegamento dos participantes e ausência de informações referentes aos protocolos utilizados na prática clínica (Quadro 1). No entanto, o cegamento deste tipo de intervenção (exercícios ativos de deglutição) é sem dúvida algo muito improvável de ser executado, principalmente diante de propostas terapêuticas tão distintas (cuidados habituais versus exercícios terapêuticos). O reduzido número de estudos e o baixo número amostral limitaram as análises do tamanho do efeito, inviabilizando o estudo de subgrupos e de correlação. Em conjunto estas limitações reduzem a força de evidência.

A qualidade da evidência (baseado no sistema GRADE) resume os julgamentos realizados para o corpo de evidência presente na metanálise e na descrição narrativa da revisão sistemática. A qualidade da evidência foi julgada como baixa em função do risco de viés nos estudos individuais e também pelas questões relacionadas à imprecisão dos resultados. As justificativas para cada julgamento constam em maiores detalhes no Quadro 2.

**Quadro 2:** Sumário dos Achados para os estudos incluídos na presente revisão sistemática

População: pacientes com câncer de cabeça e pescoço						
Contexto: ambulatorial						
Intervenção: exercícios profiláticos de deglutição						
Comparação: cuidado usual						
**Desfechos**	**Nº de participantes analisados (Nº de estudos)**	**Estudos incluídos**	**Heterogeneidade estatística (*I^2^* )**	**Tamanho de Efeito (IC95%)**	**Qualidade da evidência (GRADE)**	**Interpretação**
**Functional Oral Intake Scale (FOIS)** ^a^	33 (2)	^(11,20)^	0%	DMP	⨁⨁◯◯ **BAIXA** ^c,d^	Evidência de baixa qualidade sugere que exercícios profiláticos de deglutição reduzem a progressão de disfagia, medido pela escala FOIS
				1,27 (0,74 a 1,80)		
**Progressão de Disfagia, avaliado utilizando a escala FOIS** ^b^	165 (4)	^(11,18-20)^	NA	Dois estudos demonstram benefício e dois ausência de efeito		

a Avaliação da qualidade da evidência apenas para os estudos incluídos na metanálise^(11,20)^;

b Avaliação da qualidade da evidência para todos os estudos incluídos na revisão sistemática^(11,18-20)^ em síntese narrativa, conforme recomendações^(41)^;

Abordagem GRADE para avaliar a qualidade da evidência;

c Graduado para baixo em um nível por risco de viés nos estudos individuais devido á ausência de mascaramento para aferição de desfecho subjetivo;

d Graduado para baixo em um nível por imprecisão devido ao amplo IC95%. Dois estudos relatam resultados favoráveis à intervenção, enquanto outros dois relatam nulidade, provavelmente por baixo poder estatístico devido ao pequeno número de participantes analisados. Além disso, o número total de pacientes incluídos em todos os quatro estudos foi inferior a 200, o que gera dúvidas em relação à confiabilidade dos resultados em termos de precisão estatística considerando o Tamanho Ótimo da Informação^(42)^

Legenda: NA: não se aplica. Médias representam o valor pós-tratamento de cada grupo; DMP = representa a diferença entre grupos nas médias de pós-tratamento; IC95% = intervalo de confiança de 95%; FOIS: escala funcional de ingestão por via oral.

## CONCLUSÃO

Com base nas evidências apresentadas, é admissível supor que pacientes com CCP podem apresentar efeito positivo sobre a ingesta oral ao realizar exercícios profiláticos de deglutição quando comparados aqueles que não realizaram tal medida terapêutica ao longo da radioterapia. No entanto, a baixa qualidade da evidência e o restrito detalhamento das condutas utilizadas nos protocolos assistenciais, justificam a execução de novos estudos.

## Material Suplementar

Este artigo acompanha material suplementar.

Legenda S1 Medidas de prevenção da progressão da disfagia em pacientes com câncer de cabeça e pescoço submetidos a radioterapia: Uma revisão sistemática com meta-análise.

Este material está disponível como parte da versão online do artigo na página: https://www.scielo.br/j/codas
